# MicroRNA as an Important Target for Anticancer Drug Development

**DOI:** 10.3389/fphar.2021.736323

**Published:** 2021-08-25

**Authors:** Zhiwen Fu, Liu Wang, Shijun Li, Fen Chen, Kathy Ka-Wai Au-Yeung, Chen Shi

**Affiliations:** ^1^ Department of Pharmacy, Union Hospital, Tongji Medical College, Huazhong University of Science and Technology, Wuhan, China; ^2^ Hubei Province Clinical Research Center for Precision Medicine for Critical Illness, Wuhan, China; ^3^ St. Boniface Hospital Research Centre, Winnipeg, MB, Canada

**Keywords:** microRNA, anticancer therapeutics, drug target, MiRNA mimics, antagomirs, oncomirs, tumor suppressor miRNAs

## Abstract

Cancer has become the second greatest cause of death worldwide. Although there are several different classes of anticancer drugs that are available in clinic, some tough issues like side-effects and low efficacy still need to dissolve. Therefore, there remains an urgent need to discover and develop more effective anticancer drugs. MicroRNAs (miRNAs) are a class of small endogenous non-coding RNAs that regulate gene expression by inhibiting mRNA translation or reducing the stability of mRNA. An abnormal miRNA expression profile was found to exist widely in cancer cell, which induces limitless replicative potential and evading apoptosis. MiRNAs function as oncogenes (oncomiRs) or tumor suppressors during tumor development and progression. It was shown that regulation of specific miRNA alterations using miRNA mimics or antagomirs can normalize the gene regulatory network and signaling pathways, and reverse the phenotypes in cancer cells. The miRNA hence provides an attractive target for anticancer drug development. In this review, we will summarize the latest publications on the role of miRNA in anticancer therapeutics and briefly describe the relationship between abnormal miRNAs and tumorigenesis. The potential of miRNA-based therapeutics for anticancer treatment has been critically discussed. And the current strategies in designing miRNA targeting therapeutics are described in detail. Finally, the current challenges and future perspectives of miRNA-based therapy are conferred.

## Introduction

When researchers first discovered miRNAs in 1993, they did not realize the importance of these miRNAs. Because the first miRNAs gene Lin-4 found from the *C. elegans* that controls the timing development in its life cycle is considered to be unique in the *C. elegans*. (Lee et al., 1993). However, hundreds of miRNAs were found in different species including mammals by traditional cloning and bioinformatics methods, which attracted the attention of scientists in various fields, especially for let-7 (Reinhart et al., 2000). Up to present, a total of 38,589 miRNAs have been recorded in miRBase (v222018, www.mirbase.org), an online miRNA database. MiRNA is a single chain non-coding endogenous RNA with a length of around 22 nucleotides, which is a post-transcription regulatory factor. It plays an important regulatory role mainly by inhibiting mRNA translation or reducing the stability of mRNA. More than 90% of miRNA is in the region of encoding protein genes or introns of the gene, and few of them are in the exon region of the gene. miRNAs located in intergenic regions have independent promoter elements, while those located in coding protein genes can share specific promoter elements with host genes in addition to their own independent promoters (Bartel, 2009).

**FIGURE 1:**
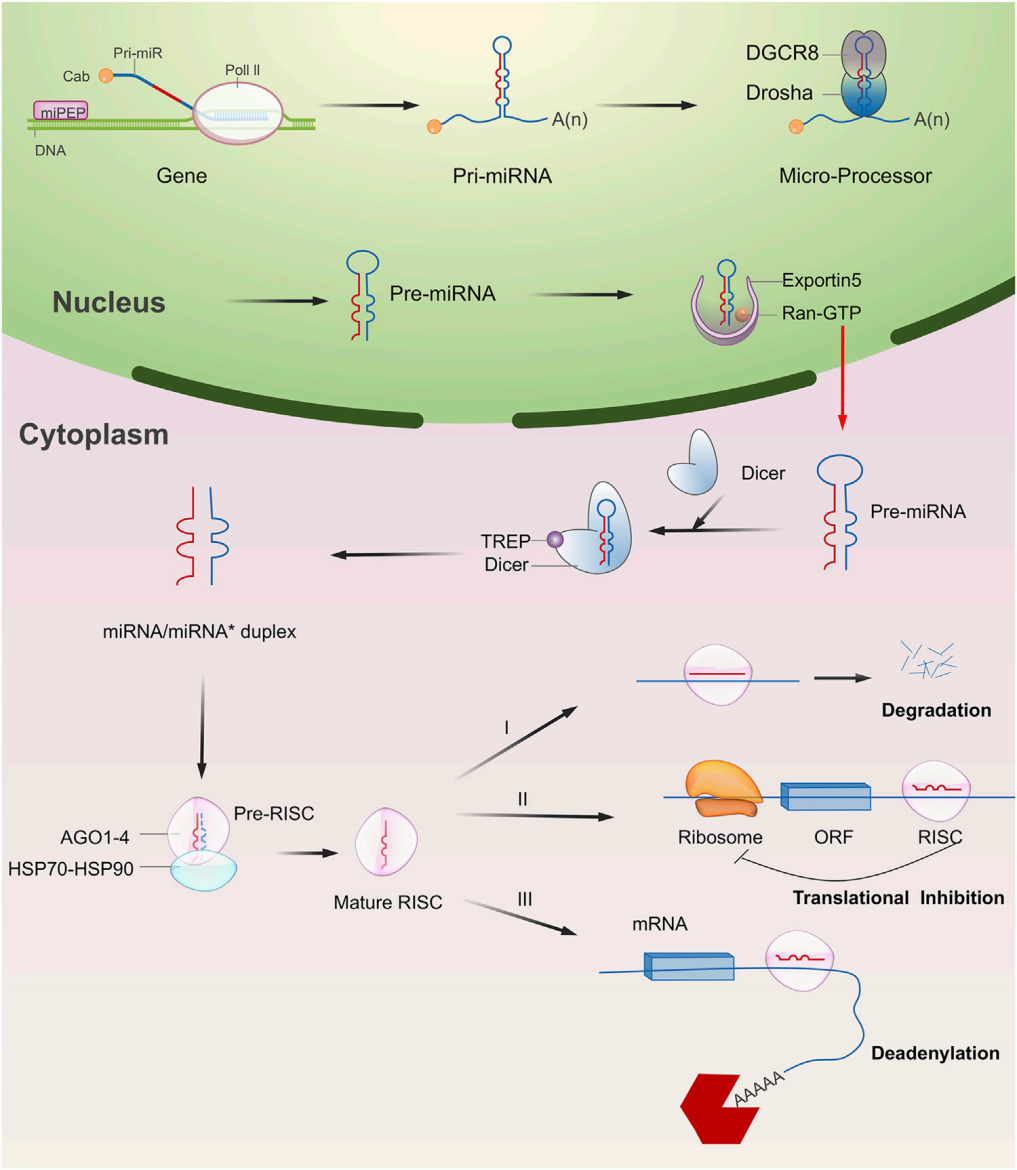
Overview of miRNA biogenesis and functions. Typically, miRNA begins with a transcription to generate the pri-miRNA. It was next processed into pre-miRNA with hairpin structure by Drosha and DGCR8 enzymes (microprocessor complex). The pre-miRNA is then exported to the cytoplasm and forms a double stranded miRNA under the processing of Dicer enzyme. A single stranded RNA that is cleaved from the double stranded miRNA is transported and assembled into a protein complex composed of Argonaute to form a RNA induced silencing complex (RISC). In most cases, RISC binds to target mRNAs to induce translational repression, deadenylation, and degradation.

The biosynthesis pathway of miRNA in animal cells is a complex process starting from nucleus to cytoplasm (Figure 1). For most of miRNAs, a primitive miRNA (pri-miRNA) is first formed from DNA sequences, and then processed into pre-miRNA with hairpin structure by Drosha and DGCR8 enzymes. Under the action of transporters composed of Ran-GTP and Exportin-5, pre-miRNA is transported to the cytoplasm and forms a double stranded miRNA under the processing of Dicer enzyme. A single stranded RNA that is cleaved from the double stranded miRNA is transported and assembled into a protein complex composed of Argonaute to form a RNA induced silencing complex (RISC), which can recognize the target gene and play an inhibitory role (Peng and Croce, 2016; Lou et al., 2018). A single miRNA could regulate the mRNA of more than one target gene, and each target gene mRNA could also be regulated by multiple miRNAs. The miRNA binding to the target mainly plays a regulatory role by post transcriptional inhibition of mRNA translation, or through a cleavage or degradation of mRNA (Wu, 2020a).

Although most of the miRNAs are located in the cytoplasm, some of them are located in the nucleus, directly regulating the transcription process at the DNA level (Pu et al., 2019). For example, miRNA-373 could up-regulate the expression level of E-cadherin after binding to the E-cadherin promoter (Wang et al., 2018a). MiRNA-mediated gene regulation is a basic post transcriptional regulatory pathway in human beings, which regulates 90% of protein-coding genes and participates in many cell biological processes (Makarova and Kramerov, 2007; Czimmerer et al., 2013). In cancer cells, mature miRNAs were found to play a crucial role in the cancer pathogenesis as an oncogenic or tumor suppressor agent because imbalance of miRNA regulation seem to be markedly associated to cancer cell proliferation, invasion, migration and metastasis, as well as apoptosis (He et al., 2020). The restoration of abnormal miRNA alterations in cancer cells using miRNA mimics or antagomirs could normalize the gene regulatory network and signaling pathways, and even reverse the phenotype (He et al., 2020; Otoukesh et al., 2020). Therefore, miRNA-based therapy provides a promising anti-cancer strategy for cancer therapy and miRNA could be also regarded as a good target for the development of anticancer drugs. In this review paper, we will summarize the latest research on the miRNA-based therapeutics for cancer and the development of anticancer drugs targeting miRNA.

## MicroRNA and Cancers

**FIGURE 2:**
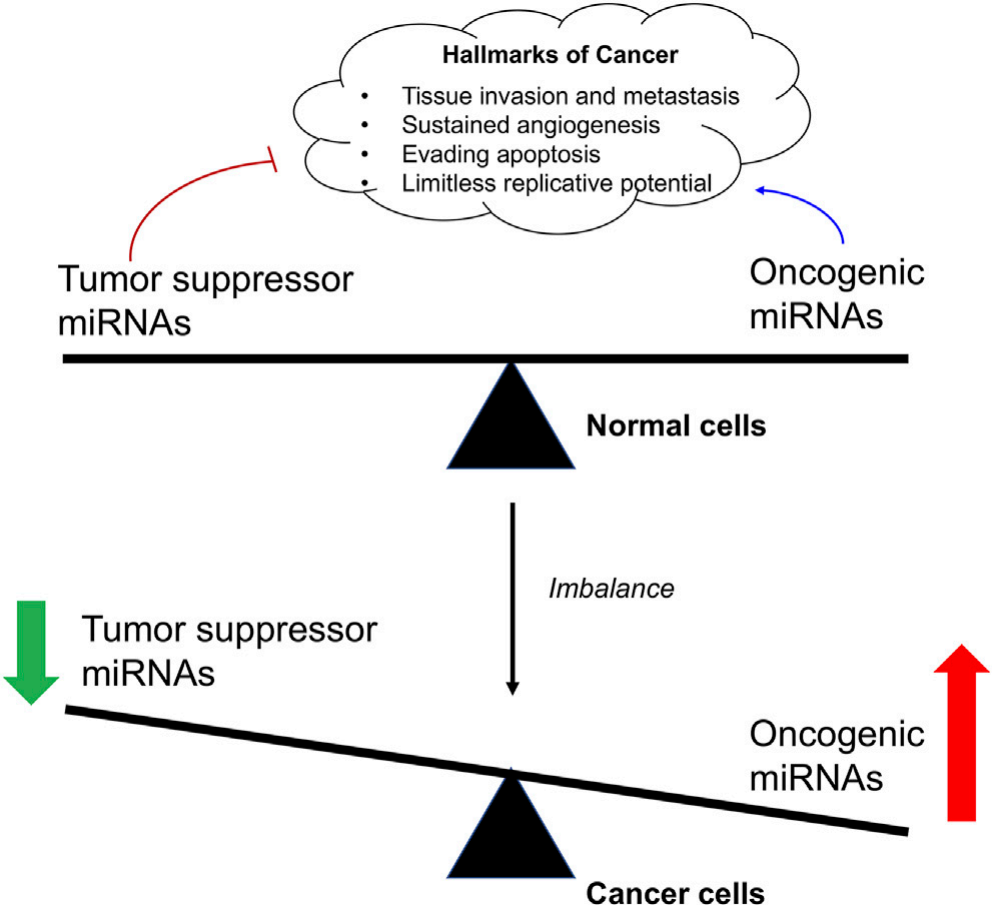
The roles of oncogenic miRNAs aand tumor suppressor miRNAs.

Cancer is a genetic disease characterized by the uncontrollable cell proliferation and apoptosis with the tumor suppressor gene mutation (He et al., 2020). Since the emergence of recombinant DNA technology, to identify the underlying mutated genes that contributes to the development of a cancer has been the central goal of cancer research. For example, the transcription factors (myc genes), Src-family, epidermal growth factor receptor (EGFR), and Raf kinase have been performed extensive researches in cancer research (Elbadawy et al., 2019; Parkin et al., 2019). In recent years, emerging evidence has shown that miRNA is also closely related to the occurrence of various types of cancers (Song and Meltzer, 2012; Chen et al., 2015a; Takasaki, 2015; Gao et al., 2018). Studies have shown that a single miRNA may bind to up to 200 targets with different functions, including transcription factors, receptors and vectors. MiRNA may control about 30% of human mRNA expression involved in cell growth, differentiation and apoptosis (Figure 2). Moreover, the expression of some types of miRNAs is found to be significantly different between normal tissues and tumor tissues, suggesting its important role in tumor occurrence, development, invasion, and metastasis (Gaur et al., 2007; Lee et al., 2008).

Based on their differential roles in the regulation of mRNA in cancer cells, miRNA are divided into oncogenic miRNAs, also known as oncomiRs, and tumor suppressor miRNAs (Table 1). For examples, the miR-15a and miR-16 were identified as the first tumor-suppressive miRNA, which negatively regulate bcl-2 (Cimmino et al., 2005). Bcl-2 is an anti-apoptotic gene, which is overexpressed in a variety of tumors, including leukemia and lymphoma (Warren et al., 2019). Therefore, the deletion or down-regulation of these two miRNAs leads to the increase of bcl-2 expression and promotes the occurrence of leukemia and lymphoma. Let-7, as one of the first identified miRNAs, is found to inhibit the expression of oncogene Ras (Johnson et al., 2005; Chirshev et al., 2019). About 15–30% of human tumors contain Ras mutation, it would cause cell transformation when the Ras mutation is activated with increased protein expression of bcl-2 (Kapoor et al., 2020). The analysis of 21 patients with different types of tumor showed that the expression of let-7 decreased significantly in twelve lung cancer patients, but only partially decreased in other tumor patients. *In vitro* tissue culture test also showed that the transient increase of let-7 in human lung cancer cells could inhibit the proliferation of cancer cells, which also indicated that let-7 might be a tumor suppressor gene in lung tissue (Takamizawa et al., 2004; Huang et al., 2020). It is also interesting to note that overall miRNA expression is lower in cancer cells than in normal cells. This suggests that a subgroup of miRNAs play a role in tumor inhibition, and the loss of their function may promote tumor occurrence. He et al. reported that three miRNAs encoded by miR-34 (miR-34 a-c) were found in human tumors by *in vivo* and *in vitro* tests, which were induced in p53 dependent cell cycle stress response or DNA damage (He et al., 2007). Among them, miR-34a is located on chromosome 1p36, and the deletion of this region could be observed in a variety of tumors (such as neurofibroma). The abnormal expression of these miRNAs down-regulates a variety of cell cycle regulatory genes, such as anti-apoptotic factor Bcl-2, resulting in cell cycle arrest and even apoptosis. Therefore, miR-34 plays an important role in the regulation of cell proliferation and apoptosis and may be used as a tumor suppressor gene.

**TABLE 1:** Aberrant expression of miRNA in cancers.

Tumor suppressor miRs (Down-regulation)		Reference	OncomiRs (Up-regulation)		Reference
miR-1	bladder, colon, lung, breast, liver, prostate, gastric cancer	Yoshino et al. (2011), Hudson et al. (2012), Reid et al. (2012), Liu et al. (2015a), Han et al. (2017)	miR-10b	esophageal cancer, gastric cancer	Tian et al. (2010), Wang et al. (2013a)
miR-7	pancreatic and colorectal cancer	Xu et al. (2014a), Xia et al. (2018)	miR-17	Neuroblastoma	Fontana et al. (2008)
let-7	breast, lung, colon, ovarian cancer	Esquela-Kerscher et al. (2008), Chirshev et al. (2019)	miR-21	Breast, colon, pancreatic, lung, prostate, liver and stomach cancer	Chan et al. (2005), Volinia et al. (2006), Zhu et al. (2007)
miR-9	ovarian cancer	Tang et al. (2013)	miR-23b	Renal cancer	Liu et al. (2010)
miR-15a	chronic lymphocytic	qeilan et al. (2010)	miR-27a	Prostate cancer	Fletcher et al. (2012)
miR-16 family	leukemia, prostate cancers, gastric cancer	Kang et al. (2015), Pekarsky and Croce (2015)	miR-100	Myeloid leukemia, glioma	Ng et al. (2010)
miR-18a	colorectal cancer	Humphreys et al. (2014)	miR-155	Lymphoma, leukemia, breast, colon, lung, pancreatic, thyroid brain cancer, diffuse large B-cell lymphoma	Jiang et al. (2010), Ling et al. (2013)
miR-25	prostate cancer	Zoni et al. (2015)	miR-222	osteosarcoma, glioma, breast cancer, follicular thyroid carcinoma, digestive system carcinoma	Quintavalle et al. (2012), Chen et al. (2013a), Matsuzaki and Suzuki (2015)
miR-27a	prostate cancer	Scheibner et al. (2012)	miR-296	Brain tumors	Würdinger et al. (2008)
miR-29 family	nasopharyngeal carcinoma, lung cancer, cervical	Sengupta et al. (2008), Garzon et al. (2009), Ugalde et al. (2011)	miR-301	Breast cancer	Shi et al. (2011)
miR-30b	laryngeal carcinoma	Li and Wang, (2014)	miR-372	Testicular tumors	Voorhoeve et al. (2006)
miR-31	breast cancer, lung adenocarcinoma	Barrett-Lee (2009), Hou et al. (2016)	miR-375	Gastric cancer	Xu et al. (2014b)
miR-33 family	chronic myelogenous leukemia, colon carcinoma, breast cancer lung metastasis, osteosarcoma	Ibrahim et al. (2011), Thomas et al. (2012), Lin et al. (2015)	miR-378	Breast carcinoma	Lee et al. (2007)
miR-34 family	breast, lung, colon, kidney, prostate, bladder, pancreatic, bone and lung cancer, and melanoma	Bommer et al. (2007), Fujita et al. (2008), Saito et al. (2015)	miR-519a	Hepatocellular carcinoma, breast cancer	Shao et al. (2015), Tu et al. (2016)
miR-124	intrahepatic, bladder, colorectal and lung cancer, osteosarcoma, neuroblastoma, glioma	Huang et al. (2011), Kato et al. (2014), Taniguchi et al. (2015)	miR-675	Colorectal cancer	Tsang et al. (2010)
miR-126	non-small cell lung cancer cells, breast, thyroid, liver, colorectal cancer, osteosarcoma	Sun et al. (2010), Wang et al. (2015), Wen et al. (2015), Xiong et al. (2015)	miR-182	Melanoma	Segura et al. (2009)
miR-128	glioblastoma, hepatocellular carcinoma, acute lymphoblastic leukemia	Zhang et al. (2009), Wuchty et al. (2011), Shi et al. (2012)	miR-483	esophageal cancer, prostate cancer	Ma et al. (2016), Yang et al. (2017)
miR-145	esophageal squamous cell carcinoma, colon carcinoma, gastric cancer, neuroblastoma	Kano et al. (2010), Ibrahim et al. (2011), Gao et al. (2013)	miR-9	Breast cancer, cervical cancer, leukemias	Selcuklu et al. (2012), Chen et al. (2013b), Azizmohammadi et al. (2017)
miR-193b	breast cancer, pancreatic ductal adenocarcinoma	Gambari et al. (2016), Yang et al. (2016)	miR-132		
miR-198	hepatocellular carcinoma	Tan et al. (2011)			
miR-204	neuroblastoma, glioma	Bao et al. (2013), Xia et al. (2015)			
miR-205	prostate cancer	Gandellini et al. (2009)			
miR-206	breast cancer	Chen et al. (2015b)			
miR-302	breast and liver cancer	Wang et al. (2013b), Liang et al. (2013)			
miR-335	breast cancer	Tavazoie et al. (2008)			
miR-383	medulloblastoma	Li et al. (2013)			
miR-449	gastric cancer, non- small cell lung cancer	Bou Kheir et al. (2011), Luo et al. (2013)			
miR-493	colon, lung cancer	Okamoto et al. (2012), Gu et al. (2014)			
miR-504	hypopharyngeal squamous cell carcinoma	Kikkawa et al. (2014)			
miR-545	pancreatic ductal adenocarcinoma, lung cancer cells	Du et al. (2014), Song et al. (2014)			
miR-596	oral squamous cell carcinoma	Endo et al. (2013)			

Alternatively, oncogenic miRNAs that promote tumorigenesis with a highly expressed in tumor tissues have also been identified. The discovery of miR-17-92 provided the first functional evidence of such an oncomiR (Gruszka and Zakrzewska, 2018). In B-cell lymphoma and cell lines, the pri-miRNA and mature miRNAs of miR-17-92 were found to be significantly overexpressed (Mihailovich et al., 2015). *In vivo* examination confirmed that enhanced miR-17-92 expression would promote the formation of myc-induced B cell lymphoma and the incidence rate of lymphoma is faster and higher. Furthermore, the expression of miR-17-92 was found to be regulated by myc protein. Myc activates its expression by directly binding to miR-17-92 site on chromosome 13 to down-regulate the expression of transcription factor E2F1 protein, which could induce apoptosis. When a single member of miR-17-92 family was induced to co-express with c-myc, it did not promote the formation of tumor, indicating that the tumorigenic effect of miR-17 family may be caused by the interaction of all members of miR-17 family (Fuziwara and Kimura, 2015). All above suggest that miR-17-92 gene cluster is a potential human oncogene. Another oncomiR, miR-21, is found to be a significant up-regulation in several types of solid tumors, such as lung, breast, prostate, and malignant glioma, supporting its oncogenic role in cancer pathogenesis (Meq, 2013; Amani-Shalamzari et al., 2020; Shakeri et al., 2021). When miR-21 inhibitor was transfected into gastric cancer cell line HEK-293, the proliferation of cancer cells was inhibited, and the apoptosis of cancer cells was induced. It also increased the proportion of G1/S phase of cancer cells, which made the cells to be more sensitive to radiotherapy and chemotherapy (Zhang et al., 2011a). In animal models, when the nude mice were injected the MCF-7cells transfected with oligonucleotides complementary to miR-21, the tumor volume was significantly decreased as 50% compared with that of control groups, and the inhibition effect on tumor growth lasted for 2 weeks (Si et al., 2007). Some studies have also revealed that miR-483 family could significantly inhibit the expression of PDGFB and directly down regulate the proliferation, migration and other malignant behaviors of human umbilical vein endothelial like cells. It was also showed that miR-483 family was shown to down-regulate the phosphorylation of Akt protein in PI3K/Akt signaling pathway after negatively regulating PDGFB (Bertero et al., 2013).

From the above, the rise of miRNA research has made scientists gradually realize that miRNA plays an irreplaceable role in the complex molecular network of oncogenes and tumor suppressor genes. The expression of miRNAs in tumors could be reduced, deleted or increased, and its expression changes are related to gene deletion, translocation, amplification or virus infection. As miRNA is a regulatory molecule in the process of gene expression and protein translation, and it plays a pivotal role in the regulation of tumor occurrence. With the breakthrough of important theories on cancer pathogenesis and the solution of difficult problems in diagnosis and treatment, it will be possible to effectively control and treat cancer in extensive research in the future.

## Potential of microRNA-Based Therapeutics for Anticancer Treatment

**FIGURE 3:**
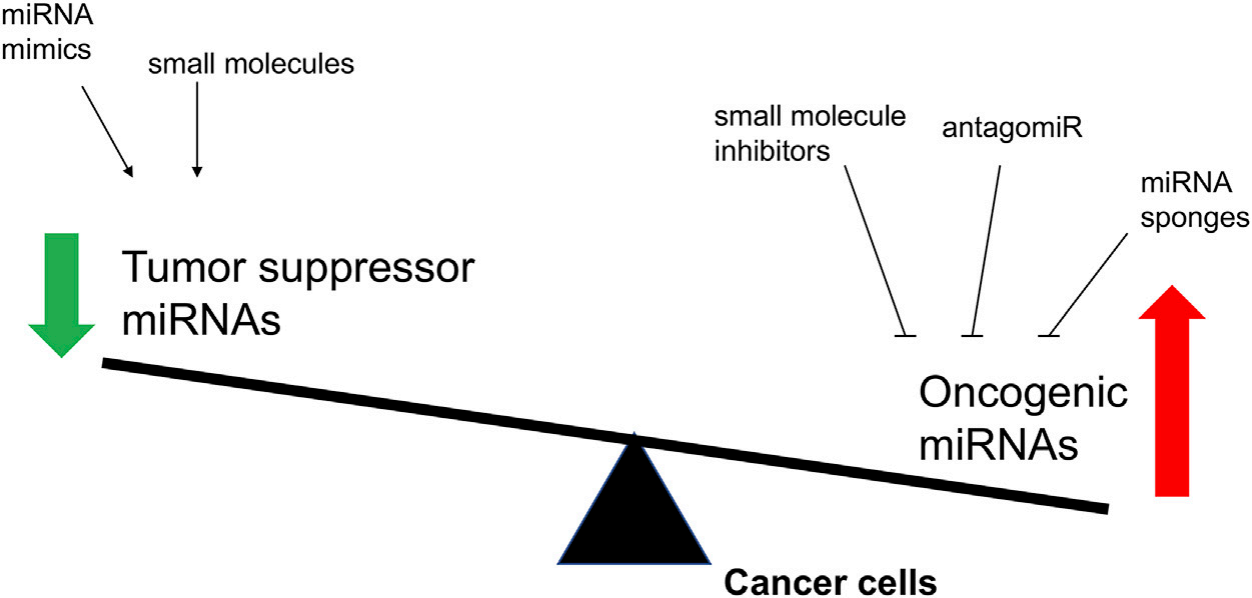
The overview of proposed strategies to regulate the biological activity of miRNAs involved in cancer. The objectives of these molecular interventions are the downregulation of oncomiRNAs or the restoring of tumor suppressor miRNAs.

As the imbalance of miRNA expression level is associated with tumorigenesis, restoring miRNA function and inhibition of overexpressed miRNAs in cancer represent the two major approaches to develop miRNA-based cancer therapies (He et al., 2020) (Figure 3). Restoring miRNA function usually applied the miRNA mimics and some small molecules, which could enhance the function of endogenous miRNAs and restore the expression of tumor suppressive miRNAs, while inhibition of overexpressed miRNAs included the small molecule inhibitors, antagomiRs, and miRNA sponges, that specifically target oncomiRs which are overexpressed in cancer cells.

### Restoring miRNA Function in Cancer

Since those miRNAs play the role as tumor suppressors, restoration of reduced tumor suppressor miRNA function to normal level by miRNA mimics represents a promising cancer treatment strategy (To et al., 2020). Indeed, the gene therapy methods were applied to restore the gene function in cancer cells before the discovery of miRNAs. However, it achieved limited success because of the limitations of DNA plasmids and viral vectors (Roma-Rodrigues et al., 2020). In recent decades, the rapid development of miRNA technology has provided an alternative tools. The size of miRNA are considerably small than that of protein so that it could permeate into cell easily by some techniques. Esquela-Kerscher et al. demonstrated that a restoration of the miRNA let-7 could observably inhibit the tumor growth on mice models (Esquela-Kerscher et al., 2008). It was the first time for this study to confirm the tumor suppressor role of let-7 and its potential to use as the targets for cancer therapy. Since then, the therapy of restoring miRNA function by miRNA mimics has rapidly gained interest. The miRNA mimics used for restoring miRNA function are summarized in Table 2. Among them, the let-7 and miR-34 families were two members to be the most studied.

**TABLE 2:** Restoring miRNA function by miRNA mimics in cancer.

Target miRNAs	Cancer types	Reference
let-7	lung cancer	Trang et al. (2010), Trang et al. (2011)
miR-34	lung cancer, prostate cancer	Wiggins et al. (2010), Trang et al. (2011)
miR-15	prostate cancer	Bonci et al. (2008)
miR-16	prostate cancer	Takeshita et al. (2010)
miR-145	colon cancer	Ibrahim et al. (2011)
miR-33	colon cancer	Ibrahim et al. (2011)

Studies have shown that let-7 family miRNAs inhibit well-known oncogenes, like NIRF, myc, HMGA, STAT3, and Ras, and the low expression of let-7 is found to be associated with poor prognosis in lung adenocarcinoma (Esquela-Kerscher et al., 2008). Hence, recovery of its activity may be a feasible strategy for lung cancer therapeutic. On the mouse lung cancer models, the effect of let-7 was examined using different delivery methods. The results showed that the let-7 mimics could reduce tumor growth, induce necrosis and de-repression of the direct let-7 targets Ras and CDK6 (Trang et al., 2010). As for miR-34 family, it was shown to be transcriptionally regulated by the tumor suppressor p53, and depletion or down-regulation of miR-34 has been found in several cancers (Hermeking, 2010). Through targeting CD44, miR-34a was shown to inhibit metastasis and reduce the growth of tumor (Wiggins et al., 2010). Similar with the supplement by let-7 mimics, when treated with the lipid-complexed miR-34a mimic on the mouse lung cancer models, the tumor volumes were significantly inhibited with a well-tolerated dose range (Wiggins et al., 2010). In the myc-driven liver cancer cells, the expression level of miR-26a was lower than in the normal health cells (Ji et al., 2009). With the replacement of miR-26a mimics to increase miR-26a levels, it would induce the cell cycle arrest through the inhibition of cyclin D2 and E2 (Kota et al., 2009). *In vivo* animals models, the tumor volumes were shown a sensitive response to the administration of miR-26a (Kota et al., 2009). All these above suggest that restoring miRNA function by miRNA mimics could provide a promising strategy for the cancer therapeutics.

### Inhibition of Overexpressed miRNAs in Cancer

As for those overexpressed oncomiRs in cancer cells, the suppression of oncomiRs has been widely studied for the development of novel miRNA-based therapeutics (Table 3). The main inhibitors of miRNA included the small molecule inhibitors and the complementary oligonucleotides, such as anti-miRNA oligonucleotide (AMOS), locked nuclear acid (LNA)-AMOS, antagomirs and miRNA sponge. AMOS is the first miRNA inhibitor based on the principle of complementary with target miRNA sequence to neutralize the function of miRNA (Weiler et al., 2006). AMOS, in the form of a short DNA oligonucleotide strand, specifically combines with complementary endogenous miRNA or its precursor molecules to form stable DNA:RNA, which makes the endogenous miRNA occupied by AMO instead of binding to its target mRNA. miRNA is thus degraded by nuclease to achieve the effect of inhibiting miRNA (Garbett et al., 2010). A series of modified AMOS, such as 2′-O-methyl AMOS, 2′-O-methoxyethyl AMOS and LNA-AMOS, have emerged in the follow-up studies. LNA-AMOS are modified on the structure of AMOS. In detail, it forms a rigid ring through a connection of methylene at the positions of the 2′-oxygen and 4′-position of multiple nucleotides, which is embedded in the C3 position of sugar group. LNA-AMOS are more stable than AMOS, and it has higher selectivity and sensitivity (Weiler et al., 2006). Similar to antagomirs, miRNA sponges could be applied to inhibit miRNA functions by preventing stable binding to their targets. Instead of short oligonucleotide strands, these agents are longer nucleic acids, usually DNA plasmids or transcribed RNA, with several miRNA binding motives.

**TABLE 3:** Inhibition of overexpressed miRNAs in cancer.

Target miRNAs	Cancer types	Reference
miR-10b	breast cancer, colorectal cancer	Sun et al. (2013), Song and Li (2019)
miR-132	breast cancer, lung cancer, liver cancer	Li et al. (2015), Zhang et al. (2014), Liu et al. (2015b)
miR-296	breast cancer, colorectal cancer	Savi et al. (2014), He et al. (2017)
miR-222	breast cancer, lung cancer	Shen et al. (2017), Yamashita et al. (2015)
miR-9	ovarian cancer	Tang et al. (2013)
miR-17	kidney cancer, ovarian cancer	Lichner et al. (2015), Gong et al. (2016)

For example, miR-10b is associated with the metastatic properties with a overexpression in breast and esophagus tumors. Through the inhibition of miR-10b, treatment of mice with the cholesterol-conjugated antagomir-10b resulted in significantly reduced levels of tumor volumes compared with the vehicle group. It is well known that tumor angiogenesis significantly enhances the invasion and metastasis of tumor cells (De Ieso and Yool, 2018; Wang et al., 2018b). With the development of technology for miRNA research, the relationship between miRNA and tumor angiogenesis becomes more evidently. High expression of miRNA-132 in the endothelium of human tumors could promote pathological angiogenesis (Zhang et al., 2011b). By reducing the expression of miRNA-132, 2′-O-Me modified antimiR-132 was demonstrated a significant decrease in tumor burden and angiogenesis, indicating the potential for miR-132 as a target in anti-angiogenesis therapeutics. In glioma, miR-9 was found an up-regulation compared with normal cells and could promote the tumorigenesis and angiogenesis. It suggested that miR-9 is crucial for glioma pathogenesis and can be treated as a potential therapeutic target for glioma.

## MiRNA as an Important Target for the Development of Anticancer Drugs

The investigation of drug targets has always been one of the most important contents in the research and development of new drugs. As a molecule widely involved in the regulation of gene expression, miRNA is undoubtedly an important object in the research of drug targets (Schmidt, 2014). Traditional drugs are mainly small chemical molecules targeting single protein in cancer which have certain limitations in clinical use. In contrast, miRNA has the natural characteristics of regulating multiple target genes, and is at the center of the multi-target regulatory network (Wang, 2011a). Moreover, the generation of miRNA is strictly regulated by signal pathway, which involves different important enzymes. MiRNA has such a complex and fine regulatory mechanism that the whole signal pathway has become a promising therapeutic drug or drug therapeutic targets in cancer (Vishnoi and Rani, 2017). Furthermore, miRNA-based drugs target molecules that cannot be target molecules that cannot be targeted by chemical drugs or antibody drugs, which is expected to make a breakthrough in diseases with poor efficacy of traditional drugs, especially cancer (Yu et al., 2020). Therefore, as drug targets, multi-target regulatory molecular miRNA has attracted more and more attention in the research and development of new drugs used in clinic.

### Restoring miRNA by Small Molecules

The down-regulated expression of miRNA can be restored by some small molecular compounds, such as the hypomethylating agents (Galm et al., 2006). Decitabine or 5-azacytidine are two drugs for the treatment of myelodysplastic and they were found to increase the expression of several miRNAs (Lujambio et al., 2007). In addition, enoxacin was also demonstrated to promote the biosynthesis of several miRNAs. In the cell-cultured tests, an overall upregulation of miRNA expression was induced by the treatment of enoxacin (Melo et al., 2011). Moreover, enoxacin reduced the tumor growth by the upregulated expression of 24 mature miRNAs on the mice xenograft models (Melo et al., 2011). All these examples suggested the feasible role of restoring miRNA by small molecules for the anticancer therapeutics.

### Restoring miRNA by Oligonucleotides

Another more specific approach for restoring miRNA is miRNA mimics. MiRNA mimics are chemically synthesized double stranded RNA molecules which regulate the function of miRNA by a simulation of endogenous miRNAs (Wang, 2011b). Because of the unstable status of miRNA mimics in the biological system, the core obstacle of the application is to develop an effective delivery system, like the nanoparticles, lipid emulsions, atelocollagen formulations, and adeno-associated virus. It was shown that the target delivery of miR-34a and let-7 mimics using the lipid emulsions could significantly inhibit the cancer progression on a colon xenograft mouse model (Trang et al., 2011). Using the adeno-associated virus as the carrier, the administration of miR-26a was demonstrated an inhibition of cancer cell proliferation and reducing tumor volume (Kota et al., 2009). Importantly, the strategy for restoring miRNA by the liposome-formulated miR-34 mimic (MRX34) has been developed to the clinical trials for the patients with liver cancer (Bader, 2012). The detailed information of MRX34 would be discussed in the subsequent sections.

### Inhibiting miRNA by Oligonucleotides

Recent decades, the most frequently used approaches to block the function of miRNA are belongs to antisense oligonucleotides (ASO) and miRNA sponges (Roberts et al., 2020). The former includes the locked nucleic acids (LNAs) and antagomirs. LNA is a synthetic nucleic acid analogue containing bridged, bicyclic sugar moiety As a novel nucleotide derivative, it has attracted extensive attention in the field of pharmaceutical research and is expected to become a new breakthrough in the treatment of various diseases at the molecular level (Papargyri et al., 2020). It is a special bicyclic nucleotide derivative with one or more 2′-O, 4′-C-methylene in its structure-β-D-furan ribonucleic acid monomer, the 2′-O position and 4′-C position of ribose form oxygen methylene bridge, sulfur methylene bridge or amine methylene bridge through different shrinkage which are connected into a ring. This ring bridge locks the N configuration of furan sugar C3′- endotype and reduces the flexibility of ribose structure. Since LNA and DNA/RNA have the same phosphate skeleton in structure, it has good recognition ability and strong affinity for DNA and RNA. A higher expression of miR-21 was associated with the cancer initiation and progression of melanoma (Javanmard et al., 2020). *In vitro* studies using B16F10 cell line, a significant reduction was found in the number of transfected cells with LNA-anti-miR-21 and the transfected cells were shown an observable apoptosis. Moreover, the treatment of anti-miR-21 could inhibit the tumor growth in the xenograft mouse models (Javanmard et al., 2020).

Antagomir is a single stranded small RNA designed according to the mature sequence of microRNA and specially labeled and chemically modified (Scherr et al., 2007). It is an efficient blocker specially used to inhibit endogenous microRNA. Antagomirs often use thiophosphate instead of phosphate to covalently bind with cholesterol at the 3′-end of oligomer to prevent the complementary matching between miRNA and its target gene mRNA by competitive binding with mature miRNA *in vivo*, and inhibit the function of miRNA (Scherr et al., 2007). It has higher stability and inhibition effect *in vivo* and *in vitro*. Arefeh Kardani et al. reported that an inhibition of miR-155 in MCF-7 breast cancer cell lines was induced by the treatmen of gold nanoparticles functionalized with antagomir-155 (Kardani et al., 2020). In another study, the inhibition of miR-194 by antagomir-194 significantly reduced the proliferation of MCF-7 and MDA-MB-231 breast cancer cells (Chen et al., 2018).

As for miRNA sponge, it is another effective inhibitor of miRNA. It contains multiple miRNA binding sites (RBS) and can adsorb corresponding miRNA molecules like a sponge. After adsorption, miRNA cannot bind to its target molecules, which affects the function of miRNA (Kluiver et al., 2012). At present, it is found that the molecules that can act as miRNA sponge include long non coding RNA (lncrna) and circular RNA (circular RNA, circrna), these two RNAs can bind miRNA or compete for miRNA target molecules and play a negative regulatory role in miRNA. For example, Shu et al. developed a system to express circular inhibitors of miRNA targeting miR-223 and miR-21 as a sponge. It was shown a more potent suppression of miRNA functions than their linear counterparts for the inhibition of cancer cell growth (Shu et al., 2018).

### Small Molecule Inhibitors of microRNA

**FIGURE 4:**
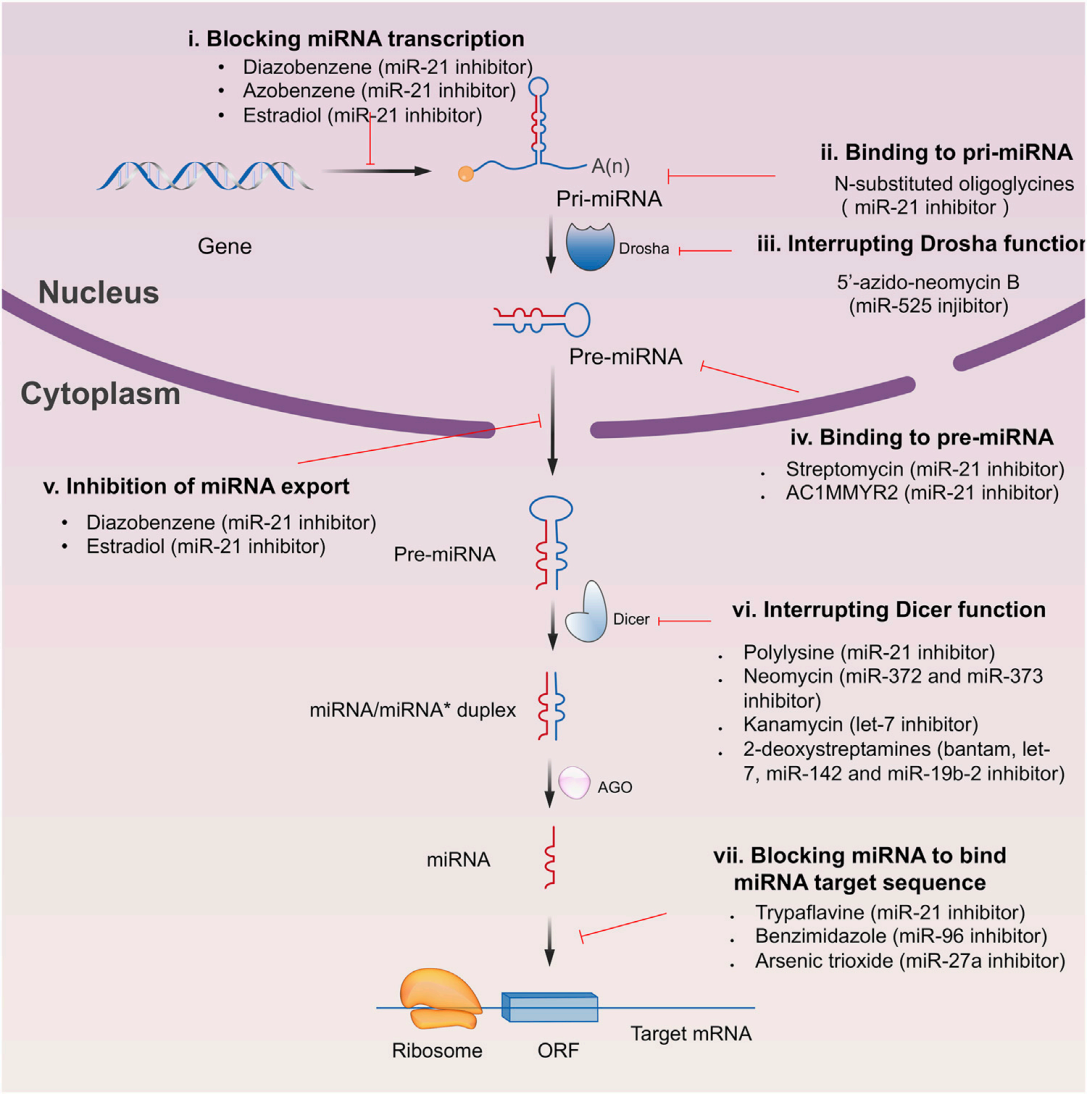
Schematic illustration of different inhibition mechanisms of miRNA specific small molecule inhibitors.

Since the biggest challenge for ASO applied in clinic is the poor cell-permeability for drug delivery, the recent trend is moving toward to the development of small-molecule drugs in the regulation of miRNA. Small molecules could cross the cell membrane by free diffusion, they modulate the function of miRNA like a microRNA mimic. Furthermore, small molecule inhibitors of microRNA are chemical compounds and thus traditional drug development can be applied (Wu, 2020b). At present, there are many kinds of miRNA specific chemical small molecule inhibitors, and their mechanisms are different. The target sites of inhibition and interference are throughout the whole process of miRNA gene expression, processing, maturation and function (Figure 4). The chemical structures of representative small molecule inhibitors were listed in Table 4.

**TABLE 4:** The summary of some representative small-molecule miRNA inhibitors.

Target miRNAs	Inhibitors	Structures	Possible mechanisms of action
miR-21	Trypaflavine	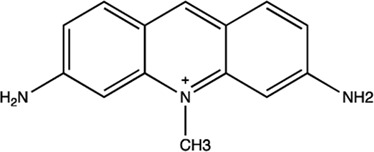	Blocking the assembly of miR-21 with Ago2 Watashi et al. (2010)
miR-21/miR-27a	Streptomycin	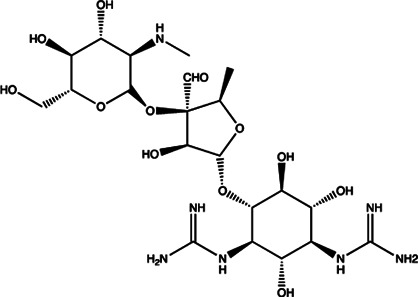	Blocking the cleavage of pre-miR-21 by Dicer Bose et al. (2012)
miR-21	AC1MMYR2	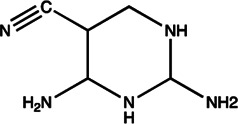	Blocking the cleavage of pre-miR-21 to produce mature miR-21 Shi et al. (2013)
miR-21	Diazobenzene	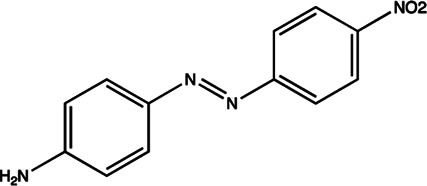	Inhibition the transcription of miR-21 gene Gumireddy et al. (2008)
miR-21	Azobenzene	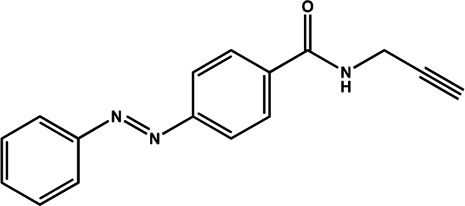	Inhibition the transcription of miR-21 gene Gumireddy et al. (2008)
miR-21	Estradiol	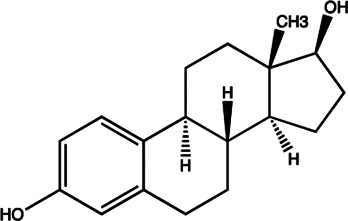	Inhibition the transcription of miR-21 gene Wickramasinghe et al. (2009)
miR-21	Polylysine	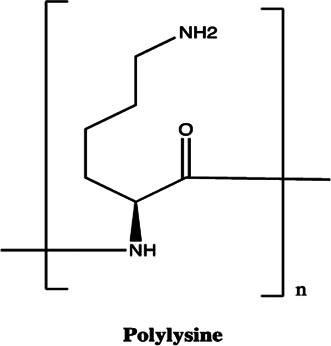	Blocking the formation of mature of pre-miR-21 by the inhibition of Dicer Watashi et al. (2010)
miR-21	4-benzoylamino-N-(prop-2-yn-1-yl)benzamides	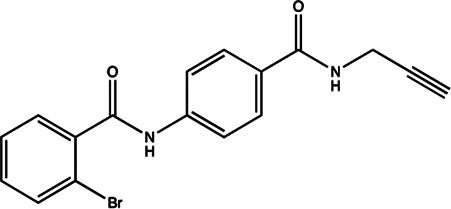	Up-regulation of PDCD4, the function target of miR-21 Jiang et al. (2015)
miR-21	Arylamide derivatives	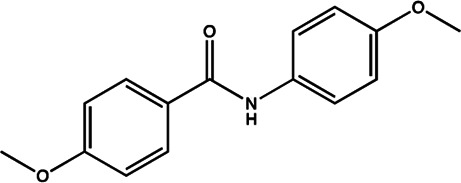	Blocking the mature of pre-miR-21 Naro et al. (2015)
Let-7/miR-27a	Kanamycin A	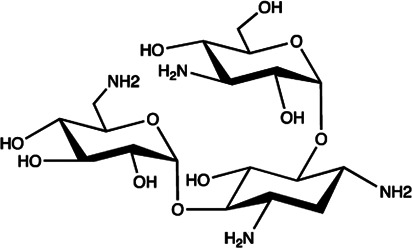	Binding to pre-let-7 and blocking the function of Dicer Davies (2008)
Let-7	2-DOS Compound 1	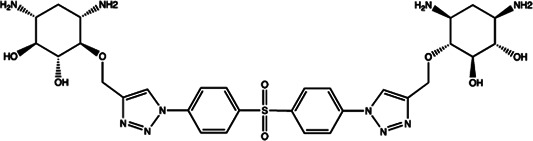	Binding to pre-let-7 and blocking the function of Dicer Davies (2008)
Let-7	2-DOS Compound 2	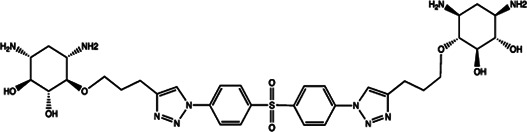	Binding to pre-let-7 and blocking the function of Dicer Davies (2008)
Let-7	2-DOS Compound 3	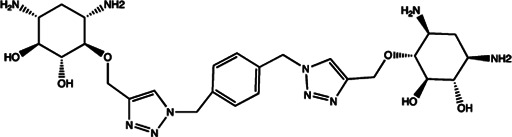	Binding to pre-let-7 and blocking the function of Dicer Davies (2008)
Let-7	2-DOS Compound 4	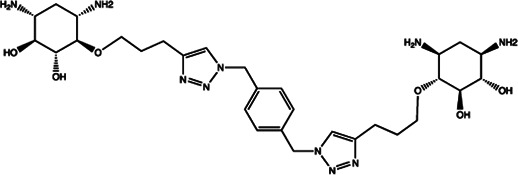	Binding to pre-let-7 and blocking the function of Dicer Davies (2008)
Let-7	2-DOS Compound 5	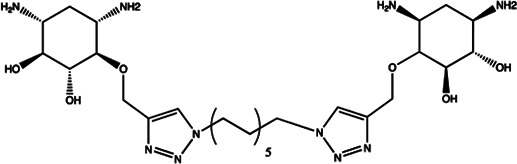	Binding to pre-let-7 and blocking the function of Dicer Davies (2008)
Let-7	2-DOS Compound 6	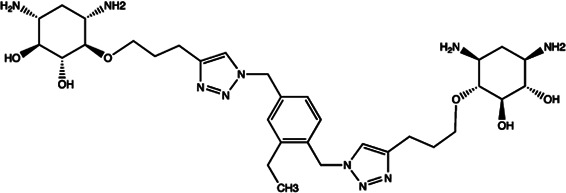	Binding to pre-let-7 and blocking the function of Dicer Davies (2008)
Bantam	2-DOS Compound 7	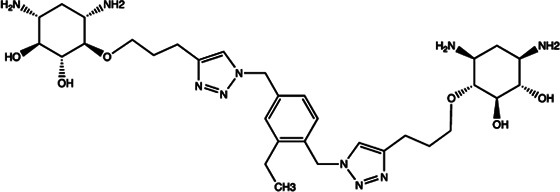	Binding to pre-bantam and blocking the function of Dicer Davies (2008)
miR-142	2-DOS Compound 8	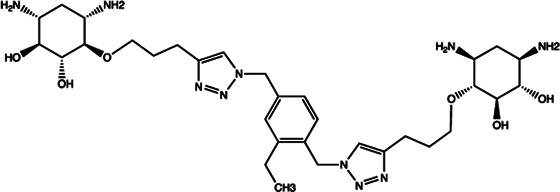	Binding to pre-miR-142 and blocking the function of Dicer Davies (2008)
miR-19b-2	2-DOS Compound 9	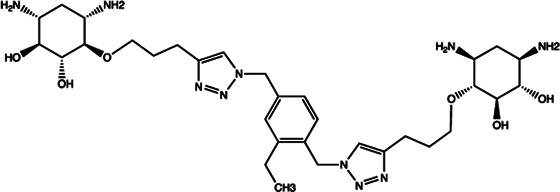	Binding to pre-miR-19b-2 and blocking the function of Dicer Davies (2008)
miR-122	NSC 158959	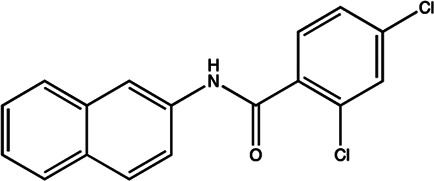	Inhibition of the transcription of miR-122 Young et al. (2010)
miR-122	NSC 5476	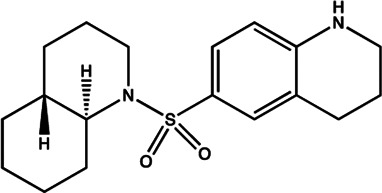	Inhibition of the transcription of miR-122 Young et al. (2010)
miR-96	Benzimidazole	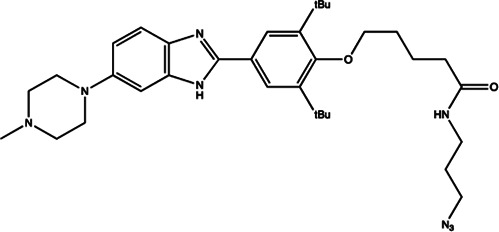	Up-regulation of FOXO1, the function target of miR-21 Velagapudi et al. (2014)
miR-1	2-methoxy-1,4-naphthalenequin	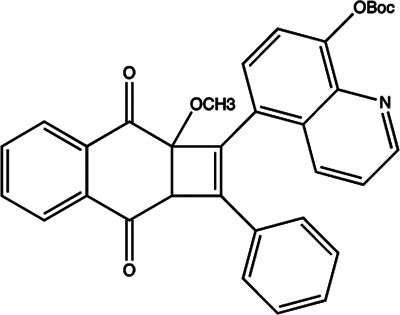	Down-regulation the expression level of miR-1 Tan et al. (2013)
miR-27a	Arsenic trioxide	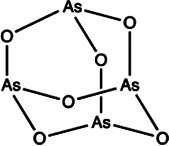	Down-regulation the expression level of miR-27a Zhang et al. (2016)
miR-27a	Neomycin	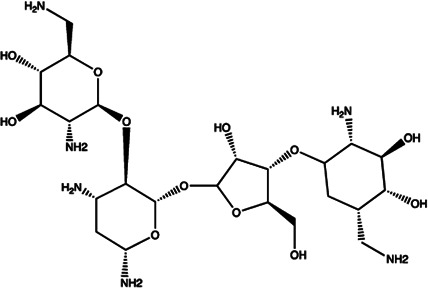	Blocking the mature of miR-27a by the inhibition of Dicer Bose et al. (2013)
miR-27a	Amikacin	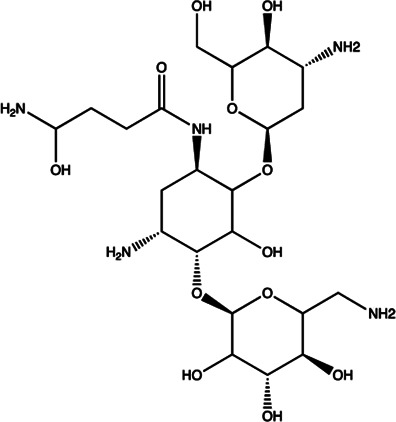	Blocking the mature of miR-27a by the inhibition of Dicer Bose et al. (2013)
miR-27a	Tobramycin	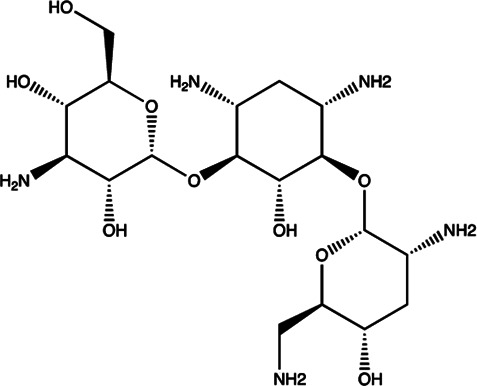	Blocking the mature of miR-27a by the inhibition of Dicer Bose et al. (2013)
miR-525	5″-azido-neomycin B	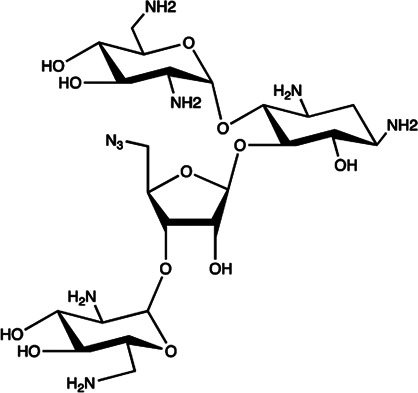	Binding to the processing site of Drosha to block the generation of pre-miR-525 Childs-Disney and Disney (2016)
miR-21	N-substituted oligoglycines	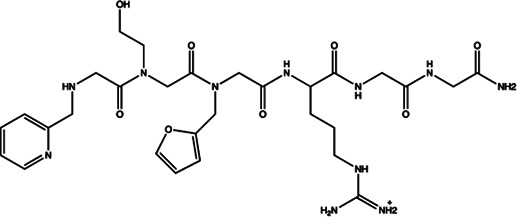	A specific ligand binding with pri-miR-21. Diaz et al. (2014)

For example, miR-21 is one of the tumor-associated microRNAs (oncomiR) that was discovered earlier and recognized. It has been confirmed in a variety of tumor cells, including breast cancer, ovarian cancer, colon cancer, pancreatic cancer, thyroid cancer, *etc*. There is high expression in cancer which are closely related to the occurrence and development of tumor (Volinia et al., 2006; Tetzlaff et al., 2007; Asangani et al., 2008; Pan et al., 2010). At present, there are abundant literatures on the chemical small molecule inhibitors. Trypaflavine (TPF), a small molecule compound, was reported by Jiang Research Group in 2010, which significantly down-regulated the expression level of miR-21 (Watashi et al., 2010). Further experiments showed that TPF could inhibit the formation of RISC by blocking the assembly of miR-21 and Argonaute 2 (ago2) protein, leading to the down-regulation of the expression level of miR-21. Davies et al*.* found that kanamycin A could inhibit the expression of let-7 by binding to pre-let-7 and interfering with Dicer (Davies and Arenz, 2006). In addition, experiments showed that the inhibition rate reached 69 ± 3% after 2 h with the treatment of 100 μM of kanamycin A.

MIR-122 is a liver specific miRNA, which is highly expressed in the liver, accounting for about 72% of the total miRNA in adult liver. It is one of the earliest miRNAs with tissue-specific and high abundance expression (Girard et al., 2008). At present, it has been found that miR-122 plays an important role in regulating liver physiological functions such as the growth cycle of hepatocytes and fat metabolism (Esau et al., 2006). It also plays a key role in the occurrence and development of liver diseases such as acute and chronic liver injury, liver cirrhosis, alcoholic hepatitis, liver tumor and hepatitis C virus (HCV) infection (Jopling et al., 2005). Deiters’s group performed the research work on the discovery of small-molecule inhibitors of miR-122 and they successfully obtained two small-molecule inhibitors (NSC 158959 and NSC 5476) with specificity towards miR-122 (Young et al., 2010). Its target may be resulted from the transcription of miR-122 gene to pri-miR-122. As for miR-1, it is highly expressed in skeletal muscle cells, which has been proved to regulate the formation of skeletal muscle cells and the development of muscle and is closely related to the development of heart (Thum et al., 2008; Wystub et al., 2013). Using 4-naphthalenequinone as the basic skeleton, dozens of derivatives were obtained by photocyclization reaction (Tan et al., 2013). The 2-methoxy-1,4-naphthalenequin, which exerted specific inhibitory effect on miR-1, was screened out from these compounds. It was confirmed that 2-methoxy-1,4-naphthalenequin could significantly down-regulate the expression level of mature miR-1 in cells. However, the specific mechanism of this compound remains to be further studied.

### Clinical Research Progress of miRNA-Related Drugs

In recent 20 years, the field of miRNA has made rapid progress and made great achievements in all directions. The three main discoverers, Victor Ambros, Gary Ruvkun and David Baulcombe won the Lasker Basic Medicine Award in 2008 and became hot candidates for the Nobel Prize in physiology and medicine for many years. At present, cooperated with academic laboratories, several pharmaceutical companies were launched miRNA clinical researches for anticancer therapeutics.

MRX34, a liposomal injection of miRNA-34a, developed by Mirnarx Therapeutics, Inc., entered the Phase I clinical trials in 2013 to evaluate the safety in patients with primary liver cancer or other selected solid tumors or hematologic malignancies (ClinicalTrials.gov, NCT number: 01829971) (Zhou et al., 2019). miRNA-34a is generally downregulated and acted as tumor suppressor in most of cancer cells by affecting more than 20 oncogenes to induce the cell apoptosis and cell cycle arrest (Welch et al., 2007). It has been shown that an increase of miRNA-34a in cancer cells would significantly inhibit the cell proliferation, suggesting a potential therapeutic strategy for cancer treatment (Yu et al., 2014). The AODNS is not permeable to enter cell by diffusion, a liposomal nanoparticle was thus employed the to carry the miRNA-34a for cancer treatment. The MRX34 is given intravenously for 5 days in a row and then 2 weeks off (total of 21 days). The trails was finally terminated as the occurrence of five immune related serious adverse events involving death of patients (Beg et al., 2017). However, the development of MRX34 indicated a feasible approach in miRNA drug discovery by using advanced formulations such as liposomal nanoparticles to increase the permeability of AODNS into the cell.

In 2014, the TargomiRs, a miR-16 based microRNA mimic, was developed by EnGeneIC Limited and underwent phase I clinical trial in patients as second or third line treatment for patients with recurrent malignant pleural mesothelioma and non-small cell lung cancer (ClinicalTrials.gov, NCT number: 02369198) (Reid et al., 2016). Several family members of miR-16 were found to be as tumor suppressors, showing a down-regulation in the broad variety of cancer cells. A restore of expression of miR-16 by a miR-16 based microRNA mimic could induce a significant inhibition of cell proliferation of some cancer cells *in vitro* models (Liu et al., 2008). As for the *in vivo* nude mice models, the miR-16 mimic need to be loaded by the nonliving bacterial minicells for the intravenous injection (Viteri and Rosell, 2018). Preliminary data revealed that the treatment of TargomiRs is controllable when the patients were exposed to five billion nanoparticles loaded with miR-16 once a week (van Zandwijk et al., 2017). The results from the phase I study were encouraging and no adverse effects were observed, therefore, TargomiR is expected to perform the phase II clinical trials in next.

MiR-155 plays a crucial role in promoting the growth and survival of the cancer cells because it is found to be highly expressed in certain lymphomas and leukemias (Poltronieri et al., 2013). In 2016, a phase I clinical trial of cobomarsen, an inhibitor of miR-155, was carried out to evaluate its safety, tolerability, pharmacokinetics and potential efficacy on the patients with cutaneous T-cell lymphoma (CTCL), chronic lymphocytic leukemia (CLL), diffuse large B-cell lymphoma (DLBCL), and adult T-cell leukemia/lymphoma (ATLL) (Anastasiadou et al., 2021). The preliminary data revealed that intratumoral injections of cobomarsen over a period of up to 15 days could improve the cutaneous lesions and without any observable adverse effects (Foss et al., 2018). Therefore, a phase II clinical trial was continued to study the efficacy and safety of cobomarsen for the treatment of CTCL and mycosis fungoides (MF) subtype in 2018 (ClinicalTrials.gov, NCT number: 03713320) (Anastasiadou et al., 2021). Although the study was terminated in December, 2020 because of some business reasons, it also encouraged to develop the microRNA as the cancer therapy.

A high expression level of miR-10b was found in glioblastoma patients by the analysis of the Cancer Genome Atlas data, indicating the oncogenic effects of miR-10b. Guessous et al confirmed that miR-10b is upregulated in human glioblastoma tissues, glioblastoma cell and stem cell lines while inhibition of miR-10b would reduce the cancer cell proliferation and inhibited invasion and migration, as well (Guessous et al., 2013). Hence, targeting miR-10b might be a good strategy for glioblastoma treatment. Furthermore, based on the critical function of anti-mir-10b in inhibition of glioblastoma growth, a clinical trial was performed for evaluating the expression Levels of microRNA-10b in patients with gliomas (clinicaltrial.gov, NCT number: 01849952). The estimated primary completion date would be May 2022. More studies are needed for confirming the effects of anti-glioblastoma in clinic.

## Challenges and Perspectives of miRNA-Based Therapy

Since developing miRNA as new drug candidates is the ultimate aim in clinical settings, the most important issue is to ensure its safety and effectiveness. Although miRNA-based therapeutics has made some progresses, there are still some barriers that limit the further application of miRNA from bench to bedside. The first limitation is delivery efficiency. The miRNA delivery systems currently used are chemically synthesized with poor cellular uptake properties (Bader et al., 2011). To make use of their medicinal effects, the miRNA should be able to across the complicated circulatory systems and the cell membranes of different tissues (Cheng et al., 2015). Since it was difficult for the miRNAs to access target cells, the second obstacle is the specificity and off-target effects of miRNA. Different from siRNA, the property of “multi-targeting” of miRNA is double-edged sword. It could help to cure the diseases by affecting as many targets correlated with pathogenesis as possible. In the meantime, it caused the off-target effects. While targeting is sequence specific rather than gene specific, it is more challenging to specifically target since the off target effect only requires partial complementary binding of miRNA and target mRNA (Singh et al., 2011). The third concern is the miRNA-induced toxicity. Studies showed that some miRNAs could transcriptionally regulate the expression of drug metabolizing enzymes, such as cytochrome P450s (CYPs) and bile acid synthase CYP7A1 (Takagi et al., 2010). Deregulating the expression of CYPs by specific miRNA may weaken the metabolism of drug to induce drug accumulation, and eventually lead to toxicity. The fourth challenge is to overcome the issue of rapid clearance in blood system (Fortunato and Iorio, 2020). For the naked nucleotide based drugs, the biggest obstacle for their function *in vivo* are the quick degradation by nuclease and the drug escape from endosome during endocytosis.

Despite the mentioned limitations, miRNA still has broad application prospects in cancer treatment. Some miRNAs may be directly related to cancer by controlling cell proliferation, differentiation and apoptosis, while others may be indirectly related to cancer by targeting oncogenes and tumor suppressor genes. Research on the role of miRNA in the occurrence and development of malignant tumor has become a hot spot. With the development of molecular biology, the detection methods of miRNA have been improved, more and more miRNAs related to cancer has been identified, and the correlation between miRNA and target mRNA has been increasingly identified. The change of miRNA expression profile is an important factor in tumorigenesis and development. Detection of miRNA in tumor patients is advantageous for tumor diagnosis, treatment and prognosis, therefore miRNA may become a new biomarker of tumor. As miRNAs are involved in the main biological behaviors of tumors by regulating target genes, new research-based therapies based on miRNAs will bring positive application prospects in the future. The clinical application of miRNA is based on high efficiency, high sensitivity and low-cost detection methods. However, the current detection methods of miRNA have certain limitations, which hinder the wide use in clinic. After overcoming these problems, miRNA detection and treatment will be expected to smoothly enter the clinic and become a new target for cancer therapeutics.
